# Supplementary material for: Influence of Graphene Sheets on Compaction and Sintering Properties of Nano-Zirconia Ceramics
Source: Materials (Basel). 2022 Oct 20;15(20):7342. doi: 10.3390/ma15207342 (PMC9611474; doi:10.3390/ma15207342)
Supplement: Supplementary file 1 [file materials-15-07342-s001.zip › Description for the Supplementary File(s) for the Figure S1 and S2.pdf]

Description for the Supplementary File(s) for the Figure S1 and S2

Examples of the resulting Archenius-type plots for the samples pure zirconia and graphenne–zirconia, shown in Figs. S1 and S2 make it possible to obtain the necessary information for calculating the sintering activation energy Q and determining the mechanisms of mass transfer. Figure S1 shows the points corresponding to the density of the material at the beginning of sintering, but not more than 4% of the initial value. It is also seen how the angle of inclination changes when graphene is introduced into the zirconium dioxide powder, which leads to a change in the activation energy.
