# Supplementary figures and images for: Influence of Graphene Sheets on Compaction and Sintering Properties of Nano-Zirconia Ceramics
Source: Materials (Basel). 2022 Oct 20;15(20):7342. doi: 10.3390/ma15207342 (PMC9611474; doi:10.3390/ma15207342)

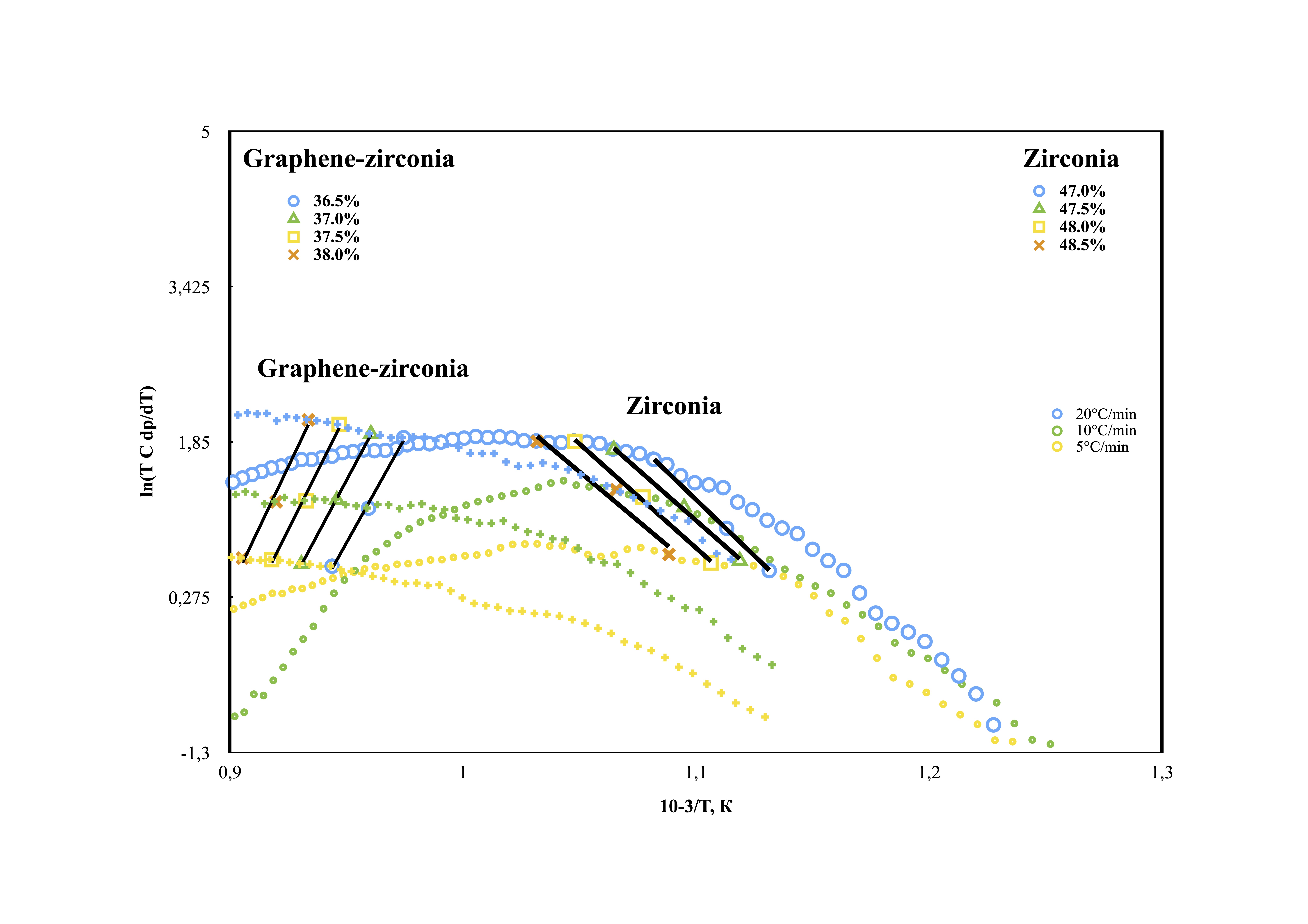

Supplement: Supplementary file 1 [file materials-15-07342-s001.zip › Figure S1.jpg]

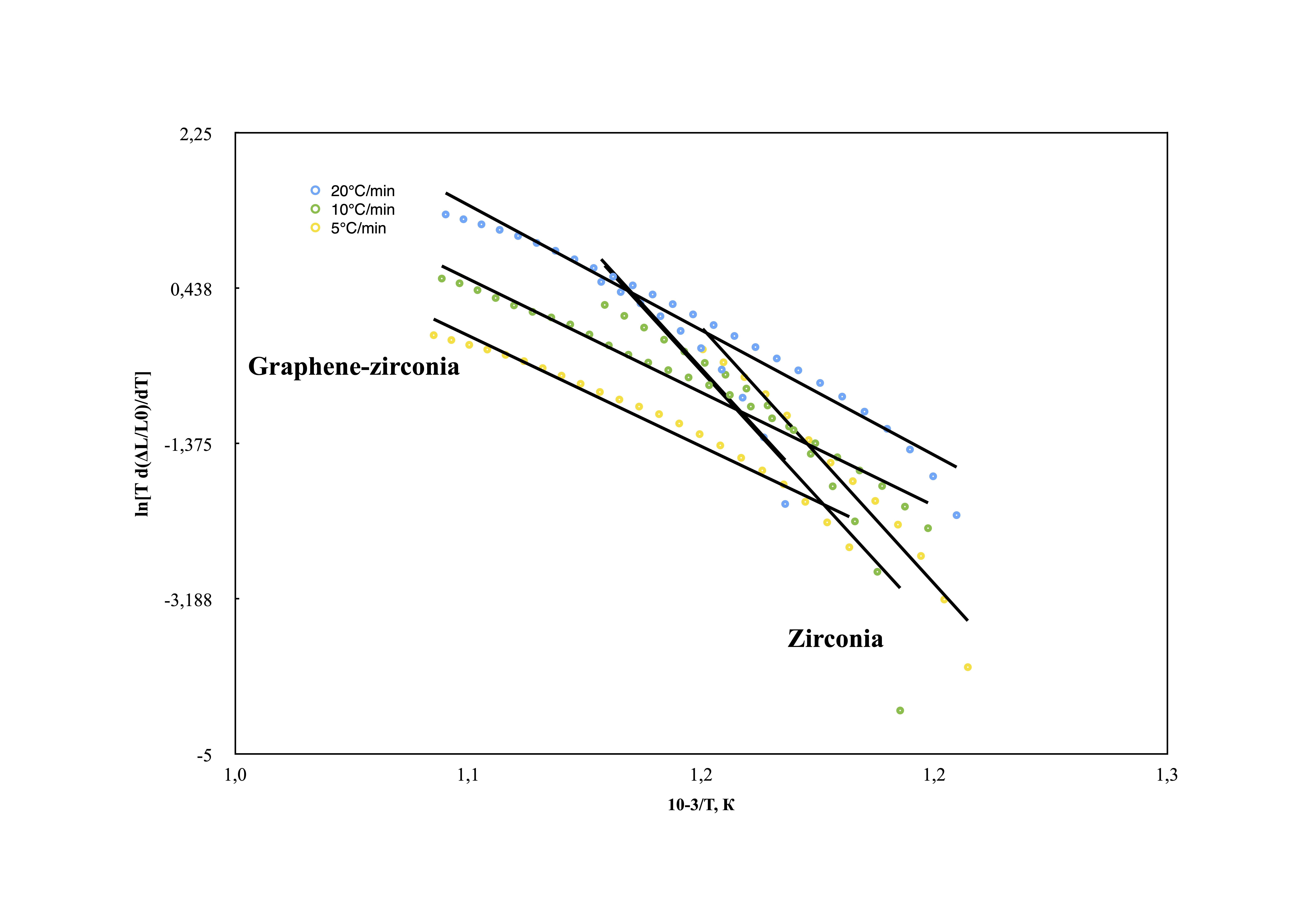

Supplement: Supplementary file 1 [file materials-15-07342-s001.zip › Figure S2.jpg]
